# The optomotor response of the praying mantis is driven predominantly by the central visual field

**DOI:** 10.1007/s00359-016-1139-3

**Published:** 2016-12-22

**Authors:** Vivek Nityananda, Ghaith Tarawneh, Steven Errington, Ignacio Serrano-Pedraza, Jenny Read

**Affiliations:** 1 Institute of Neuroscience, Henry Wellcome Building for Neuroecology, Newcastle University, Framlington Place, NE2 4HH Newcastle upon Tyne, UK; 2 Faculty of Psychology, Universidad Complutense de Madrid, Campus de Somosaguas, 28223 Madrid, Spain

**Keywords:** Optomotor response, Spatial integration, Praying Mantis, Motion detection

## Abstract

The optomotor response has been widely used to investigate insect sensitivity to contrast and motion. Several studies have revealed the sensitivity of this response to frequency and contrast, but we know less about the spatial integration underlying this response. Specifically, few studies have investigated how the horizontal angular extent of stimuli influences the optomotor response. We presented mantises with moving gratings of varying horizontal extents at three different contrasts in the central or peripheral regions of their visual fields. We assessed the relative effectivity of different regions to elicit the optomotor response and modelled the dependency of the response on the angular extent subtended by stimuli at these different regions. Our results show that the optomotor response is governed by stimuli in the central visual field and not in the periphery. The model also shows that in the central region, the probability of response increases linearly with increase in horizontal extent up to a saturation point. Furthermore, the dependency of the optomotor response on the angular extent of the stimulus is modulated by contrast. We discuss the implications of our results for different modes of stimulus presentation and for models of the underlying mechanisms of motion detection in the mantis.

## Introduction

The optomotor response is a behaviour that has been widely used to investigate insect vision (Reichardt and Wenking 1969; Pick and Buchner 1979; Reichardt and Guo 1986). It consists of whole-body movement in the direction of motion in response to perceived wide field motion (Reichardt and Wenking 1969; Kaiser and Liske 1974; Poggio and Reichardt 1976). The response has been particularly useful to investigate the spatiotemporal frequency and contrast ranges of insect visual systems and has been used to determine the contrast sensitivity functions of several insects including mantises (Reichardt and Wenking 1969; Pick and Buchner 1979; Reichardt and Guo 1986; Nityananda et al. 2015). Its dependency on a variety of visual parameters has thus been well characterized.

The optomotor response is typically triggered by optic flow across a wide area visual field. For naturally behaving insects, such optic flow would be generated by their own body movements and the optomotor response would serve as a stabilizing movement. Body movements by flying insects typically have six degrees of freedom. Three of these would related to translation (thrust, lift, sideslip) while the other three would be rotational (yaw, pitch, roll) (Balint and Dickinson 2004). Each of these distinct types of movements generates a distinct pattern of optic flow on the visual sphere (Gibson 1950; Koenderink and van Doorn 1977; Krapp and Hengstenberg 1996; Glennerster and Read 2016). The optic flow in turn leads to specific flight or motion responses from insects. For example, optic flow with radial expansions and contraction flow lines on the retina drives both the landing response of flies (Wehrhahn et al. 1981; Borst and Bahde 1986; Duistermars et al. 2007; van Breugel and Dickinson 2012; Baird et al. 2013) and defensive responses in other insects (Robertson and Johnson 1993; Tammero et al. 2004; Santer et al. 2005; Sato and Yamawaki 2014).

**Fig. 1:**
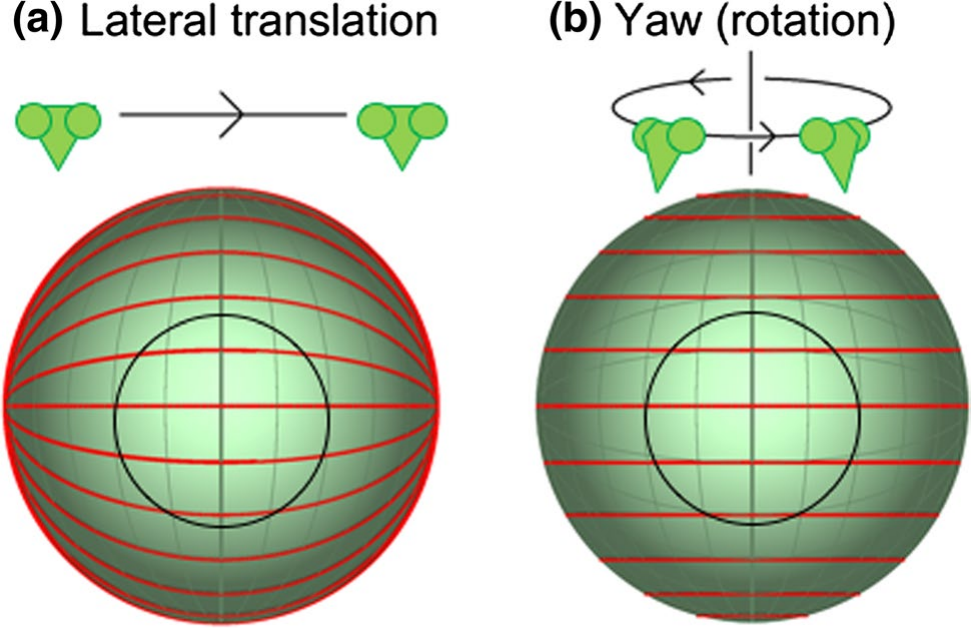
Optic flow patterns that elicit the optomotor response. Optic flow lines on the visual hemisphere generated by **a** lateral translation (sideslip) and **b** rotational motion (yaw). The visual axis is straight ahead, directly into the page. The *faint green lines* represent directions 15° apart on the visual hemisphere. *Red lines* represent the direction of optic flow on the retina. *Sketches* above each panel depict the mantis head motion that would generate such optic flow. Within the central 60° or so (*central black circle*), the flow patterns are similar for the two flow types. Flow lines, however, differ in the extreme periphery outside this circle

The stabilizing optomotor response of insects can be elicited both by the optic flow pattern generated by lateral translation, i.e. sideslip (Fig. 1a), and also by that generated by rotation about an axis perpendicular to the line of sight, i.e. yaw (Fig. 1b) (Reichardt and Wenking 1969; Pick and Buchner 1979; Reichardt and Guo 1986; Nityananda et al. 2015). To study the optomotor response in the laboratory, yaw (self-motion about the dorsoventral axis, Fig. 1b) is best simulated by presenting stimuli on a rotating cylinder around the insect (Reichardt and Wenking 1969; Pick and Buchner 1979; Reichardt and Guo 1986) while lateral translation perpendicular to the line of sight (Fig. 1a) is best simulated by presenting stimuli on a planar monitor screen (Dvorak et al. 1980; Srinivasan and Dvorak 1980; Nityananda et al. 2015). Both these approaches have been used to study the optomotor response in insects, but we know little about how or whether the optomotor response differs for the two types of flow. The answer depends critically on how much the optomotor response integrates information across the retina. The patterns of optic flow are horizontal lines of longitude for sideways translational motion (Fig. 1a) and horizontal lines of latitude for yaw rotational motion (Fig. 1b). In the vicinity of the fovea, however, the flow is very similar in the two cases (Fig. 1, within the central black circle). It differs mainly in the periphery. Thus, differentiating these two types of flow requires integrating information from the visual periphery.

The extent of spatial integration involved in the optomotor response has been surprisingly understudied. Some work has investigated vertical integration perpendicular to the direction of motion flow (Borst et al. 1995). Other studies have identified neurons responsive to motion flow in the horizontal direction and their spatial sensitivity to this motion (Hausen 1982a, b). However, what is relevant here is integration over different visual angles along the direction of motion flow (here horizontal).

**Fig. 2:**
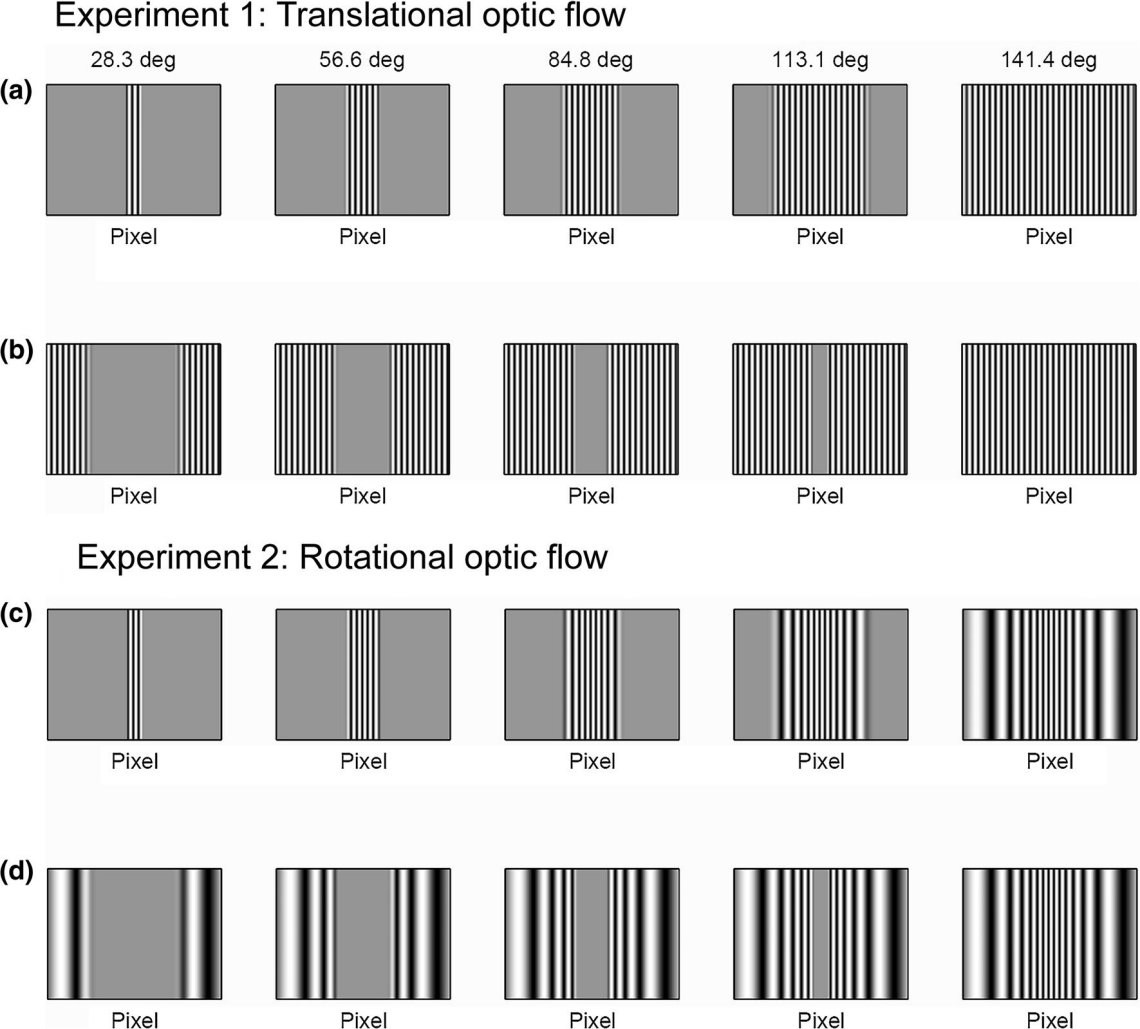
Screen previews of the central (**a**, **c**) and peripheral (**b**, **d**) stimuli. **a**, **b** Stimuli used in Experiment 1. **c**, **d** Stimuli used in Experiment 2. The number above each plot is the sum of visual degrees subtended by the gratings in each condition. Gratings in each vertical pair within an experiment subtend the same visual angle in total. Note that the same visual angle takes up more of the screen area near the edge of the screen than near the centre (see geometry in Fig. 4)

**Fig. 3:**
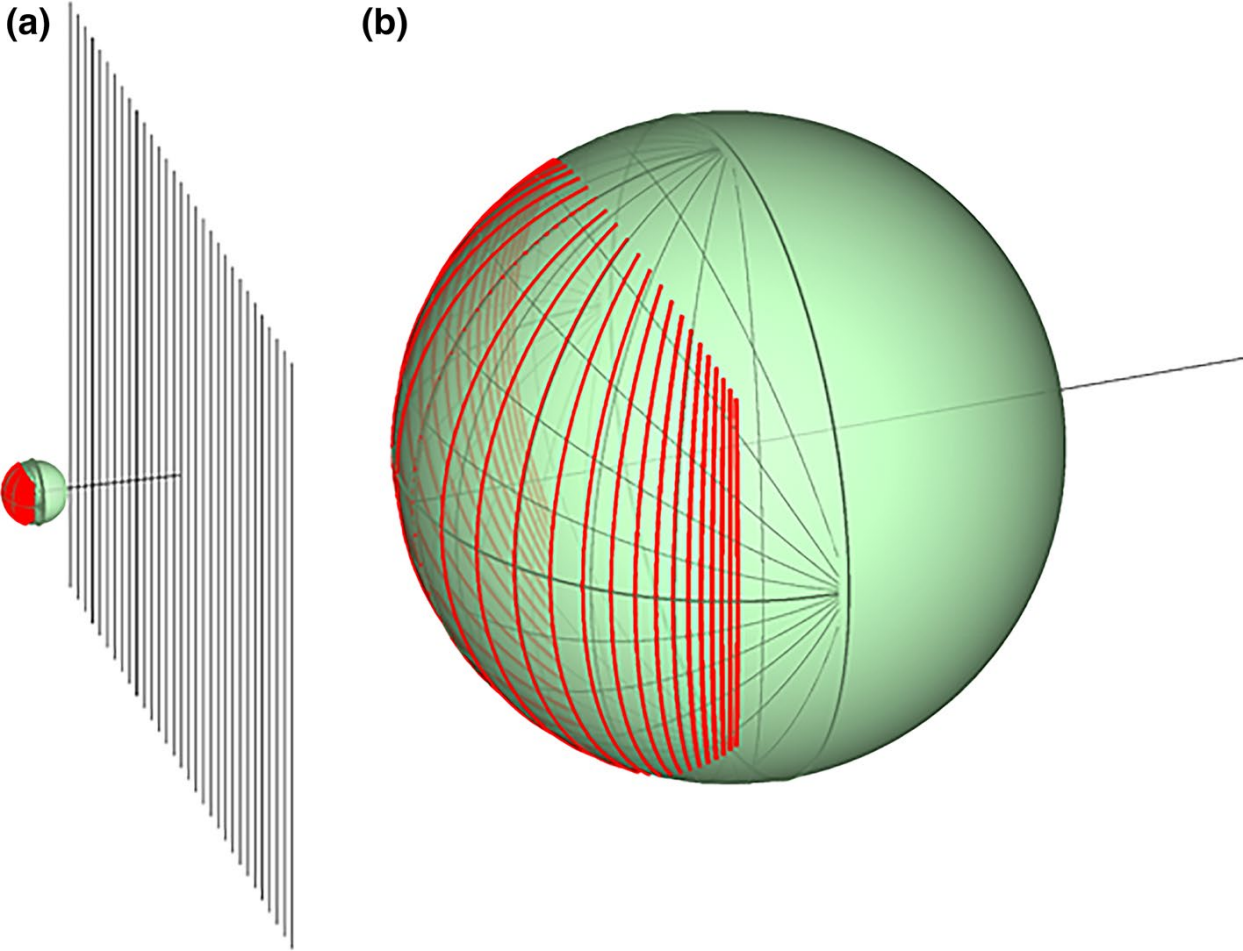
The stimulus on the mantis retina. **a** The *straight black lines* represent the peaks of a sinusoidal luminance grating on the planar monitor. The *green sphere* represents the visual field of an observer at the position of the mantis, 7 cm from the middle of the screen. The *red lines* show where each *black line* projects to on the visual field. **b** Zoomed-in version of the visual field. The *thin green lines* mark on an azimuth-longitude/elevation-longitude coordination system, lines drawn at 15° intervals, while the *thick red lines* show where the stripes of the sine grating project to. Each stripe is a line of longitude on the visual sphere, but the angle between successive stripes gets smaller as eccentricity increases. This means that the spatial frequency of the pattern increases with eccentricity. The angular speed of the motion, therefore, decreases (speed = temporal frequency/spatial frequency)

As a first step towards investigating this, we therefore designed our study to examine the relative contribution of central and peripheral regions of the visual field to the optomotor response. We presented moving luminance gratings on a planar screen and restricted the moving stimulus to central or peripheral regions of the visual field along the direction of motion. In the first condition, the gratings moved with constant speed and spatial frequency on the planar monitor (Fig. 2a, b). This simulates lateral translational motion of the insect (Fig. 1a). The flow lines are horizontal lines of longitude on the visual sphere, and the spatial frequency on the insect’s retina increases with eccentricity while the angular speed decreases (Fig. 3). In the second condition, the stimulus was designed so as to approximate a grating moving with constant speed and spatial frequency on a virtual cylindrical surface surrounding the insect (Fig. 2c, d). This simulates yaw rotational motion of the insect (Fig. 1b), where both spatial frequency and speed are constant across the retina. In both conditions, the total visual angle subtended by the stimulus was varied to see how this affected the optomotor response. By comparing the responses, we could deduce whether the central or the peripheral presentation of optic flow was more effective in eliciting the optomotor response.

**Fig. 4:**
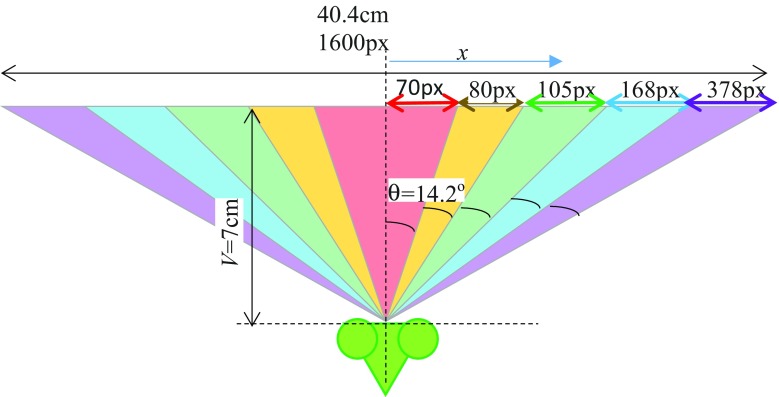
Top-down view of the mantis in front of the computer screen. The *colours* show the 5 different regions within which grating stimuli were presented (Fig. 1). We use *x* to represent horizontal position on the screen, in pixels, and *θ* to represent angular position in the visual field; tanθ = *x/V,* where *V* is the viewing distance. All marked angles are the same, i.e. 14.2°. Thus, when we consider its locations on either side of the *midline*, each region covers a total of 28.3°

This question is especially interesting in an insect such as the praying mantis which has a central foveal region of the eye where visual acuity is higher (Rossel 1979). It may be that the mantis weights movement information in this central high-acuity region more strongly than movement in the periphery, where acuity is lower. Mantises are also largely stationary ambush predators, but do show peering behaviour (Horridge 1986; Kral 2012), where they move their heads laterally to generate motion parallax information for depth computation. Such a behaviour generates predominantly translation perpendicular to the line of sight (Fig. 1a). We might, therefore, expect different results in praying mantises compared to flying insects without foveae, which are likely to experience predominantly translation parallel to the line of sight, generating radial flow, and rotational motion.

## Methods

### Animals

We ran two experiments with 6 individuals each of the species *Sphodromantis lineola* in each experiment. Each insect was stored in a plastic box of dimensions 17 × 17 × 19 cm with a porous lid for ventilation and fed a live cricket twice per week. The boxes were kept at a temperature of 25°C and were cleaned and misted with water twice per week.

### Experimental setup

As in Nityananda et al. (2015), the setup consisted of a CRT monitor (HP P1130) and a 5 × 5 cm Perspex^®^ base which the mantises held onto while hanging upside down facing the [horizontal and vertical] middle point of the screen at a distance of 7 cm. The Perspex base was held in place by a clamp attached to a retort stand and a web camera (Kinobo USB B3 HD Webcam) was placed underneath providing a view of the mantis but not the screen. The monitor, Perspex^®^ base and camera were all placed inside a wooden enclosure to isolate the mantis from distractions and maintain consistent dark ambient lighting during experiments.

The screen had physical dimensions of 40.4 × 30.2 cm and pixel dimensions of 1600 × 1200 pixels. At the viewing distance of the mantis, the horizontal extent of the monitor subtended a visual angle of 142° and the vertical extent subtended a visual angle of 130°. The mean luminance of the monitor was 46 cd/m^2^ and its refresh rate was 85 Hz. We used a Minolta LS-100 photometer to measure the luminance levels of the monitor and applied the appropriate Gamma correction (Gamma = 2.0) using the PTB function PsychColorCorrection(‘SetEncodingGamma’).

The monitor was connected to a Dell OptiPlex 9010 computer with an Nvidia Quadro K600 graphics card and operated on Microsoft Windows 7^®^. All experiments were administered by a Mathworks Matlab (2012b) script which was initiated at the beginning of each experiment and subsequently controlled the presentation of stimuli and the storage of keyed-in observer responses. The web camera was connected and viewed by the observer on another computer to reduce the processing load on the rendering computer’s graphics card and minimize the chance of frame drops.

### Visual stimulus

Visual stimuli were developed using Psychophysics Toolbox version 3 (Brainard 1997; Pelli 1997; Kleiner et al. 2007). In each trial, the stimulus was a sinusoidal grating with a constant spatial frequency of 0.1 cpd on screen, and a Michelson contrast of either 0.05, 0.20 or 1.00, moving to either left or right at a temporal frequency of 8 Hz for 5 s.

**Fig. 5:**
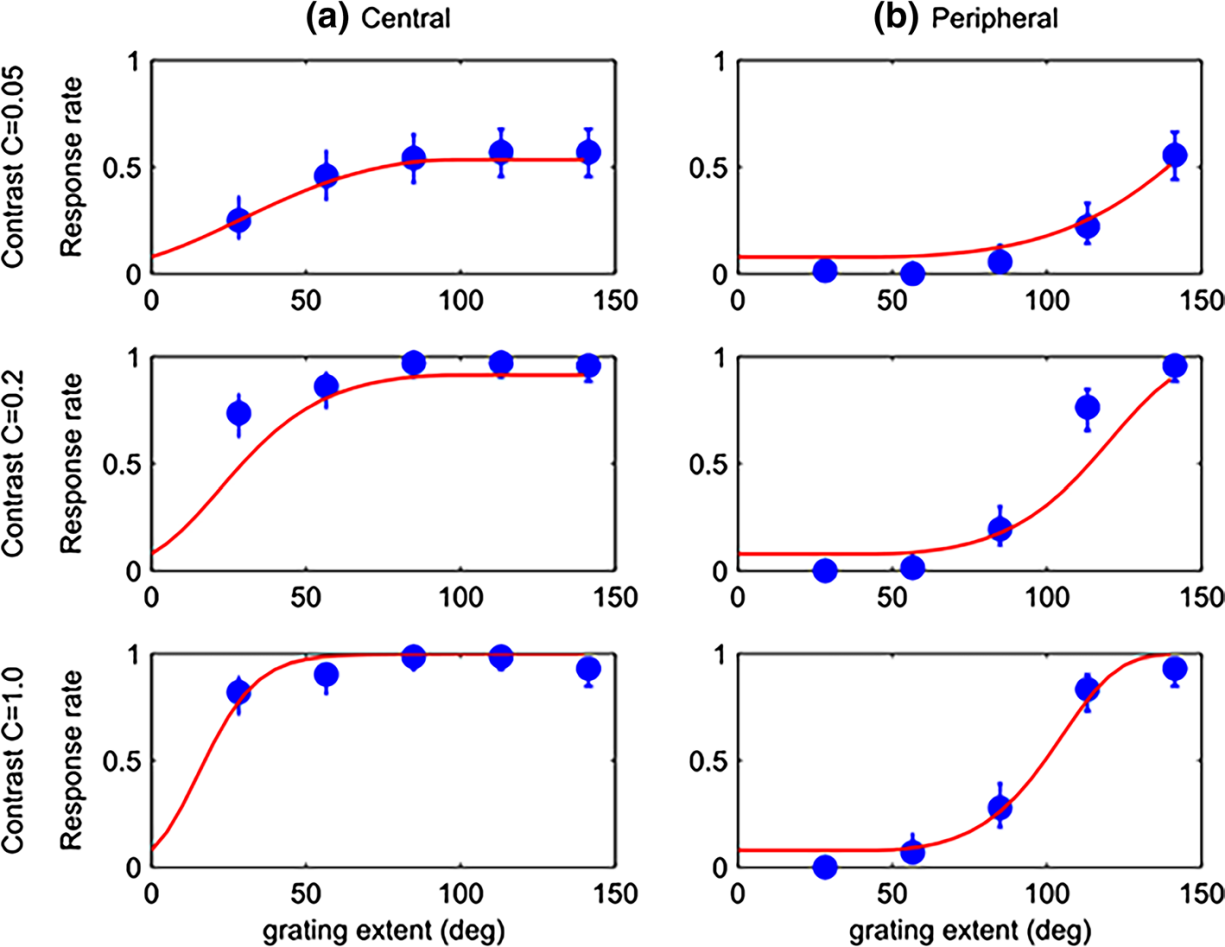
Mantis psychometric functions for Experiment 1. *Blue symbols* show the mean response rate (probability of observing the optomotor response) as a function of the angle subtended by the moving grating. *Error bars* represent the 68% confidence interval assuming simple binomial statistics (equivalent to ±1 standard error for normally distributed data). *Columns* represent responses to central (*left*) and peripheral (*right*) stimuli. *Rows* represent responses to different contrasts. The *red curves* show the results of a model with 2 free parameters which were fitted simultaneously to all 6 conditions (30 data points) (*M* = 0.064, *b* = 49°; see “Methods” section for details)

Stimuli were presented in two locations: “central” and “peripheral”. In the central condition, gratings were restricted to an extent of the central region of the visual field by multiplying luminance levels with the Butterworth window function:

$$w\left( x \right) = \frac{1}{{1 + \left( {\frac{\left| x \right|}{W/2}} \right)^{2n} }}$$

where *x* is the horizontal pixel position (relative to the middle of the screen), *W* is the window size in pixels (defined as the distance between the half-gain points) and *n* is the window function order (taken as 10 in our experiments). In the peripheral condition, on the other hand, gratings were restricted to peripheral regions of the visual field by multiplying luminance levels with the complementary function *h*(*x*) = 1 − *w*(*x*). The values of *W* used during the experiment corresponded to 5 fixed-step increments of visual degrees up to the screen width (i.e. 28.4°, 56.8°, 85.2°, 114° and 142°). As shown in Fig. 5, this corresponds to values of *W* = 140, 299, 509, 844, 1600 pixels. In the central stimulus condition, gratings thus occupied linearly increasing extents starting from the middle of the screen (Fig. 2a, c) while for the peripheral condition, gratings occupied the same total visual degrees but extending from the two sides of the screen (Fig. 2b, d).

In Experiment 1, these sine gratings were presented unmanipulated on a planar screen (Fig. 2a, b). The spatial period was a constant 53 pixels or 1.33 cm on the screen. At the viewing distance of 7 cm, this subtended an angle of 10.6° when positioned at the centre of the screen, but only 1.26° when positioned at the edge. The luminance level of each pixel on the screen was calculated as:

$$l_{1} \left( p \right) = \sin \left( {2\pi \left( {f_{\text{ps}} \times p + f_{t} \times t} \right)} \right)$$

where *f*_ps_ is the spatial frequency in cycles/px (0.0189), *p* is the horizontal pixel position relative to the centre of the screen, *f*_*t*_ is the temporal frequency (8 Hz) and *t* is the time.

In Experiment 2, we applied a non-linear horizontal transformation to simulate the presentation on a cylindrical surface. The spatial period of the stimuli was increased towards the edges of the screen, so as to subtend a constant angle of 10° at the mantis, regardless of position on the screen (Fig. 2b, c). This was done by first calculating the visual angle of each pixel relative to the centre of the screen,

$$\alpha \left( p \right) = { \arctan }\left( {\frac{p}{D \times R}} \right),$$

where *D* is the viewing distance (7 cm) and *R* is the pixel resolution (39.6 pix/cm), and then rendering luminance levels as:

$$l_{2} \left( p \right) = \sin \left( {2\pi \left( {f_{s} \times \alpha \left( p \right) + f_{t} \times t} \right)} \right)$$

where *f*_*s*_ is the spatial frequency in cpd (0.1). By applying this transformation, all grating periods subtended a constant visual angle of 10° regardless of their position on the screen.

### Experimental procedure

In both experiments, each experimental run consisted of 900 trials in which an individual mantis viewed moving gratings covering various regions of their visual field (depending on stimulus condition, see Fig. 2). An experimenter observed the mantis through the camera underneath and blindly coded the direction of the elicited optomotor response, if any. The response typically consisted of a combined movement of the entire body and head in the direction of the grating motion. The response code for each trial was either “moved left”, “moved right” or “did not move”. The mantises were shown gratings in both locations (central and peripheral) across five window sizes (Fig. 2: 28.4°, 56.8°, 85.2°, 114° and 142°) and three different contrasts (0.01, 0.05 and 1) making for a total of 30 conditions. There were 30 repeats of each condition per experiment (consisting of 15 left-moving and 15 right-moving gratings), for a total of 900 trials per mantis. The different stimuli within an experiment were presented in a pseudo-random order chosen by the computer.

In between trials, a special “alignment stimulus” was presented and used to steer the mantis back to its initial body and head posture as closely as possible. The alignment stimulus consisted of a random chequer-like pattern which could be moved in either horizontal direction by keyboard shortcuts and served to re-align the mantis by triggering the optomotor response.

### Data collection and analysis

Observer SE collected the data for the presented experiments. We tested the response rates for both central and peripheral conditions. The response rate for each stimulus condition was calculated as the proportion of trials in which the blindly coded direction matched that of the grating.

### Statistical analysis

We ran a generalized linear model with the number of correct responses as the dependent variable, using a Poisson log link function and the region of presentation (central or peripheral), visual angle subtended and contrast as predictors. We made planned comparisons between the number of correct responses for different contrasts in the central region of presentation to investigate the effect of contrast in this specific condition.

### Modelling

Our data are described well by a very simple one-dimensional model in which the efficacy of a region of visual space in driving the optomotor response declines linearly with horizontal eccentricity, until a maximum eccentricity, beyond which stimuli do not contribute to eliciting the response. Formally, we assumed that a vertical strip of stimulus at eccentricity *x* degrees would have weight *D*(*x*) in driving the optomotor response. We assumed that

1 $$D(x) = \left\lfloor {M\left( {{{1 - \left| x \right|} \mathord{\left/ {\vphantom {{1 - \left| x \right|} b}} \right. \kern-0pt} b}} \right)} \right\rfloor$$

where $\left\lfloor z \right\rfloor$ = *z* when *z* > 0 and 0 otherwise. *M* is the maximum contribution per degree, which applies at the fovea (*x* = 0°), and *b* is the bounding eccentricity beyond which stimuli no longer help drive the optomotor response. The optomotor response is also elicited more reliably by stimuli of higher contrast (Nityananda et al. 2015). We write *W*(*C*) for the weight of contrast C, normalized such that *W*(1) = 1 by definition; clearly *W*(0) = 0 (a zero-contrast stimulus cannot drive the optomotor response). In general, therefore, the total optomotor drive provided by a one-dimensional grating of a given spatial and temporal frequency is

2 $$S = \int {{\text{d}}xD(x)W(C)}$$

where *C* is the contrast of the stimulus, and the integral is taken over the stimulus. For example, for a central grating of extent *g*, extending between eccentricities ±*g*/2, *S* = *Mg*(4*b* −* g*)/(4*b*). The maximum possible signal is *S*_max_ = Mb, when the grating has an extent of 2b.

We can estimate *W*(*C*) from the data in Nityananda et al. (2015). Their Figure 2, middle bottom panel, shows the optomotor response rate as a function of contrast for full-screen gratings of the relevant spatial and temporal frequency (SF = 0.098 cycles per degree, TF = 8 Hz). The maximum response rate is 0.62 at *C* = 1. For *C* = 0.2, it is 0.39 (i.e. 63% of the maximum) while for *C* = 0.05 it is 0.21 (34% of maximum). We, therefore, estimate *W*(0.05) = 0.34 and *W*(0.2) = 0.63.

We then applied a traditional signal detection theory model. That is, we assumed that within the mantis brain, the signal *S* is subject to Gaussian noise, and the mantis is then classified as making an optomotor response if the sum of signal and noise exceeds a fixed threshold *θ*. The noise level does not need to be a free parameter in our model, since changes in the noise are already accounted for by changes in the maximum signal strength *M*. Without loss of generality, therefore, we fixed the noise variance to a value of 0.5. This means that the expected response rate is

3 $$R_{\text{pred}} = 0.5(1 + {\text{erf}}(S - \theta ))$$

where erf is the Gauss’s error function,

$${\text{erf}}\left( x \right) = \frac{2}{\sqrt \pi }\mathop \int \limits_{0}^{x} \exp \left( { - t^{2} } \right){\text{d}}t.$$

We found that good fits were obtained if the threshold *θ* was close to 1. Therefore, we did not include *θ* as a free parameter in our fitting, but fixed it at 1, meaning that the threshold is equal to sqrt(2) times the SD of the noise. We adjusted the 2 free parameters *M* and *b* so as to maximize the likelihood of observing our actual response rates *R*, assuming simple binomial statistics. Optimization was carried out by the Matlab function FMINSEARCH.

## Results

Across all contrast values in Experiment 1, mantises showed some response to central stimuli at even the lowest grating extent of 28.4° (Fig. 5, left column). This response to central stimuli increased with increase in grating extent until it saturated at a grating extent of around 85°. In contrast, when gratings were presented in the peripheral regions (Fig. 5, right column), mantises did not respond to the lower grating extents and only responded to gratings subtending angles greater than 85°. The response rates at higher grating extents gradually increased but did not clearly saturate even at the highest grating extent of 142°. If we look at the actual areas covered by stimuli on the screen for these grating extents (Fig. 2), we see that stimuli encroaching from the periphery do not elicit the optomotor response until they start overlapping with the central ~85°. Stimuli presented in the centre, however, immediately elicit optomotor responses. The mantis optomotor response thus seems to be driven solely by stimuli in the central ~85° of the visual field, and not at all by those in the periphery.

The varying contrast levels of the gratings had a significant main effect on the mantis response rates (GLM, *χ*_2_^2^ = 32.34, *P* < 0.001; Fig. 5, rows). For central presentations, mantises made an equal number of responses to contrast levels of 1 or 0.2 (GLM, pairwise comparisons, Mean difference = 0.55, *P* = 0.26). However, at a contrast level of 0.05, the number of responses was much reduced (GLM, pairwise comparisons, Mean differences [to contrast = 1, 0.2] = [−4.95, −4.40], Ps < 0.01). Interestingly, the contrast did not affect the way the grating extent affected the response rate. Thus, the response rate for gratings at all contrasts saturated at 85° independent of contrast. Similarly, gratings presented in the periphery generated no response to smaller grating extents for all contrasts. The response to peripheral stimuli also only began to show saturation for the largest grating extent at the highest contrast. This implies that the movement in the central visual field is necessary to elicit the optomotor response; lack of input in the central field cannot be compensated for by increasing contrast in the periphery.

**Fig. 6:**
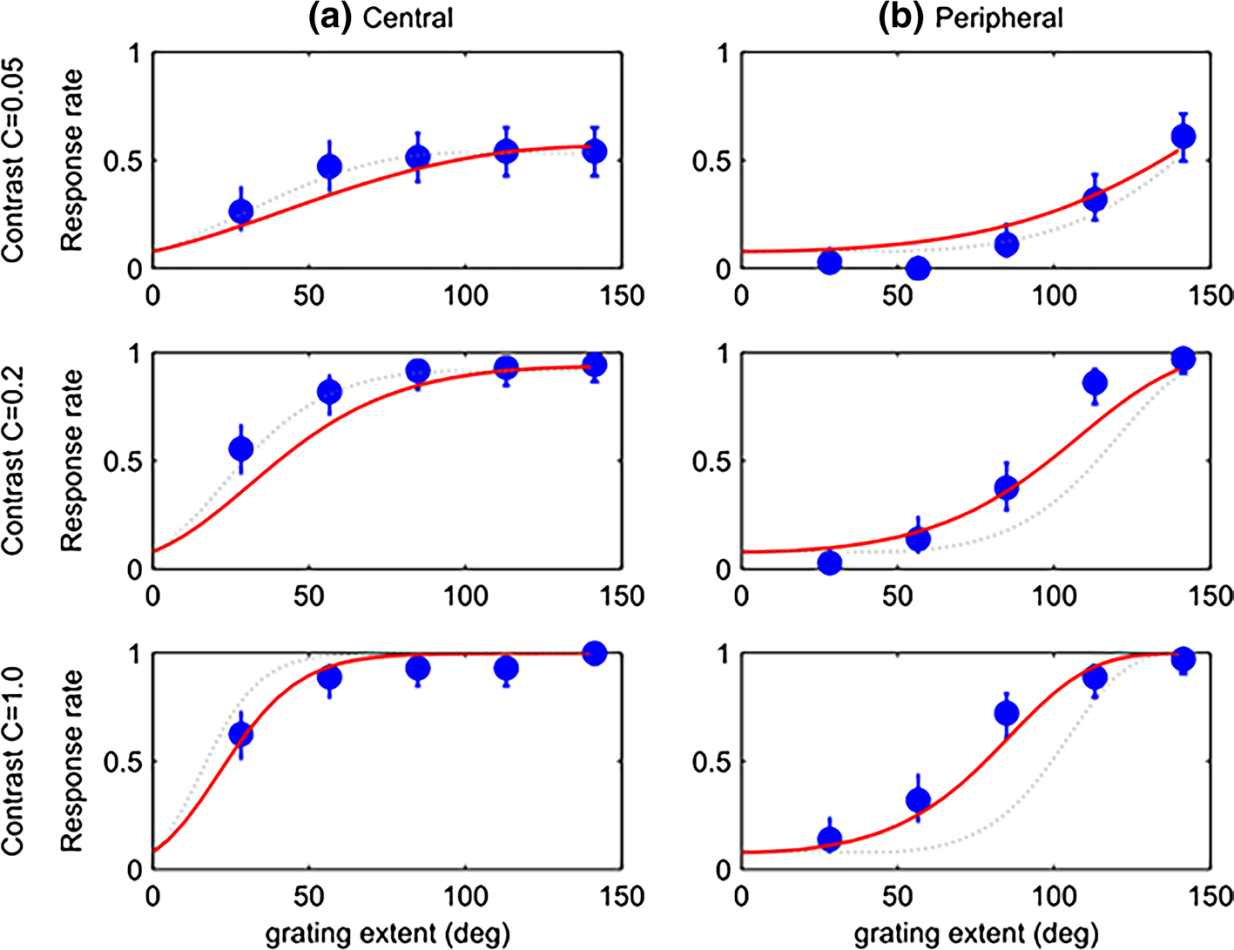
Mantis psychometric functions for Experiment 2; other details as for Fig. 3. The *red solid curves* show the results of a model with 2 free parameters fitted simultaneously to these data (*M* = 0.046, *b* = 71°); for comparison, the *dotted curves* show the Experiment 1 fits (*M* = 0.064, *b* = 49°, as shown previously in Fig. 5)

In this experiment, the stimuli were sine gratings on a planar screen. Gratings in the centre of the screen, therefore, presented lower spatial frequencies to the retina than gratings in the periphery. One possible explanation for our results might, therefore, have been that mantises have different sensitivities to the frequencies they saw in the centre compared to the periphery. To rule out this possibility, we modified the stimulus in Experiment 2. In this experiment, the stimuli were warped so that the spatial frequencies at the retina were the same across all regions of the screen, simulating presentation of a sine grating on a cylindrical screen (Fig. 2c, d). Our results from Experiment 2 were qualitatively very similar to the results described above for Experiment 1 (Fig. 6), although the response rate rose somewhat less for every increase in grating extent for central stimuli, and rose more for peripheral stimuli. This indicates that the peripheral regions of the screen were more effective in driving the optomotor response once the spatial frequencies had been adjusted as in Experiment 2. However, the central region clearly remains much more effective than the periphery, and this must genuinely reflect different sensitivity rather than the effect of spatial frequency or to translational versus rotational optic flow. Furthermore, an inspection of the videos recorded shows that they make similar optomotor responses to stimuli in both Experiments 1 and 2. This indicates that this response is fairly stereotyped and does not differ when faced with translational or rotational optic flow.

The aspects of the data described above suggested to us the model explained in the “Methods”. The red curves in Figs. 5 and 6 show the response rates predicted by such a model with just 2 free parameters fitted simultaneously to all 6 stimulus conditions (30 data-points) in each Experiment. The fitted parameters were *M* = 0.064 and *b* = 49° in Experiment 1, and *M* = 0.046 and *b* = 71° in Experiment 2. With just two free parameters plus two further parameters taken from an independent data-set published in a previous paper (Nityananda et al. 2015), this simple model does a good job of capturing the data.

**Fig. 7:**
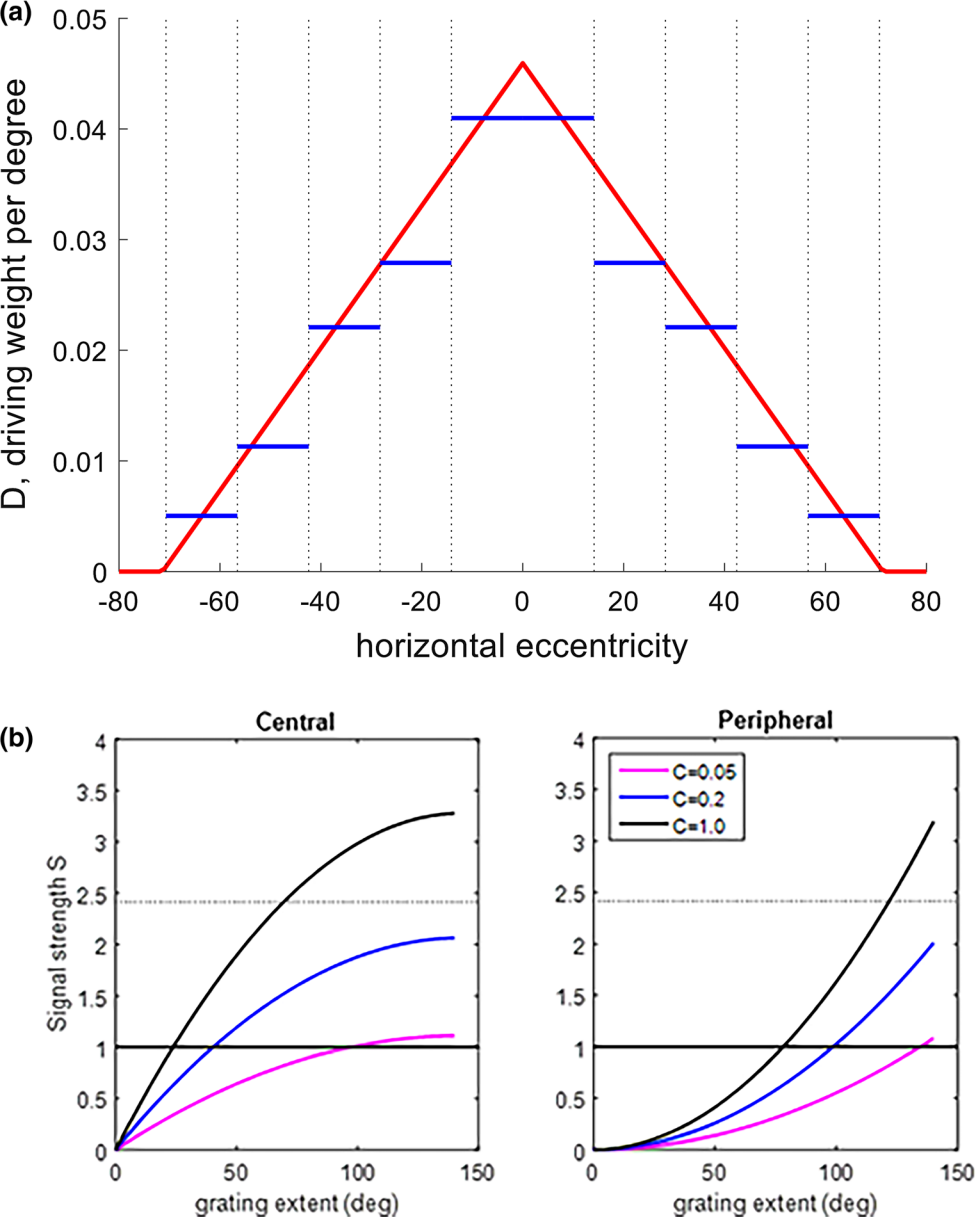
Detailed description of the model. **a** The modelled signal per unit degree contributing to the optomotor response, as a function of horizontal eccentricity. The sloping red lines depict the function *D*(*x*), Eq 1, with parameters fitted to Experiment 2, i.e. *M* = 0.046, *b* = 71°. The *vertical dotted lines* mark the boundaries of the five stimulus regions used in our model. The *horizontal blue lines* show the results of a more elaborate model in which the average signal contributed by each region was a free parameter, instead of being constrained to be the average value of the *red line* (Eq 1) in each region [fitted parameters: threshold *θ* = 0.90, average driving weight per degree for each patch = 1.16, 0.79, 0.62, 0.32, 0.14 (centre to periphery); cf values for linear model = 1.17, 0.91, 0.66, 0.40, 0.14]. **b** The modelled total signal driving the optomotor response, as a function of grating extent for central (*left*) and peripheral (*right*) locations. The curves depict the function *S*, Eq 2, for different stimulus sizes. The parameters were those fitted to Experiment 2, i.e. *M* = 0.046, *b* = 71°. The colours show the three different contrasts. The *solid horizontal line* shows the threshold, which was constrained to be equal to the SD of the noise, and the *dotted line* the threshold plus one SD. In our model, an optomotor response is recorded if the signal strength exceeds this threshold plus a random sample from a Gaussian with this SD

Figure 7 shows the model’s internal structure for the fit to Experiment 2. The sloping red lines in Fig. 7a show the weight *D*(*x*) (Eq 1) as a function of horizontal eccentricity *x*. Stimuli at the fovea are weighted most strongly in driving the optomotor response, and the weight declines linearly as a function of eccentricity towards the edge of the screen at ±71°. Figure 7b shows the resulting total signal *S* (Eq 2) as a function of grating extent for the central and peripheral conditions across different contrasts. As the grating extends from the centre (Fig. 5a), the signal initially rises steeply, but then saturates, reaching its peak when the grating covers the full screen. The half-maximal value is obtained for a grating extending across an angle *b*(2 − √2), or 42° (between ±21°). Conversely as gratings move in from the edge, the signal initially rises slowly and then accelerates. This explains why in the data (Fig. 6) little increase in response is seen when the central stimuli expand beyond the central 57°, and why the most peripheral stimuli do not elicit any response at all except at the highest contrast.

Of course, an exact linear dependence of driving weight per degree with eccentricity is almost certainly a simplification. We also fitted a more general model in which the total driving weight of each portion of grating was a free parameter, instead of being specified by integrating Eq 1. We also fitted the threshold as free parameter. The fitted weights for this more complex model are shown by the horizontal blue lines in Fig. 7a. The model again gives most weight to stimuli close to the fovea and progressively less weight to more eccentric stimuli. The fall-off is not quite linear, as can be seen by the fact that the blue lines are not exactly at the average value of the red line for each patch, but the deviation is small. Other, more elaborate models are also possible (for example, we could fit the contrast weights instead of taking them from our previous paper), but give very similar fits to our data and do not change our conclusions.

## Discussion

We tested the ability of moving gratings presented on a planar screen in either the central or peripheral regions of the visual field to drive the optomotor response in the praying mantis. We found that motion in the central regions of the visual field drives the optomotor response much more strongly than motion in the periphery. We developed a simple model with just two free parameters, which gave an excellent account of the data.

We found that a given grating extent is much more powerful at eliciting the optomotor response when presented at the centre of the screen. When gratings extend inwards from the periphery, the response continues to rise all the way up to the widest extents tested, 140°. Conversely, when gratings extend outwards from the centre, the response saturates when the grating covers around 80°; in Figs. 5a and 6a, there is no difference in the response rates for the last 3 data points. This is not simply because the response is already maximal, since the same saturation point is observed for the 5% contrast stimulus, where the response rate never rises above 50%.

All the features of our data could be well modelled simply by postulating a linear decrease in weight with eccentricity and a maximal response at the fovea. The model is not perfect; for example, in the left column of Fig. 6, it is noticeable that the modelled response rate (solid red line) for the lower contrasts continues to increase as the central grating expands right to the edges of the screen, whereas the data are flat beyond a grating extent of ~80°. Conversely, in the right column of Fig. 6, the model predicts more response to low-contrast peripheral stimuli than is observed. Nevertheless, for such a simple model, the agreement is good.

The same model structure explained the data from both Experiments, but Experiment 2 required a larger bounding eccentricity *b* (71° as compared to 49° for Experiment 1), with a correspondingly lower signal at the fovea (*M* = 0.046 vs *M* = 0.064) for a similar maximum signal (*M* × *b* = 3.3 vs 3.1). This probably reflects the stimulus. In Experiment 2, the stimulus was designed to present a constant spatial frequency at the mantis retina, and thus a constant angular speed of flow across the retina. In Experiment 1, the spatial frequency of the grating was constant on the planar monitor, meaning that the spatial frequency experienced on the mantis retina increased with eccentricity. The spatial frequency at the centre of the monitor was already towards the upper limit of mantis sensitivity (Nityananda et al. 2015), and it appears that the increasing frequency combined with the decreasing weight of the visual periphery to make the effective drive decrease even more steeply with eccentricity. By testing both types of flow (translational in Experiment 1 and rotational in Experiment 2), which are similar at the fovea but differ in the visual periphery, we confirm that the greater weight given to foveal motion does not depend on the type of flow. A limitation of our study is that we have used only a single spatial frequency, which is towards the upper limit of frequencies which elicit optomotor responses in mantises. This limit is essentially imposed by the geometry: as Fig. 2 shows, even this frequency only allows just over 1 cycle in the smallest peripheral patch. We could not therefore examine substantially lower frequencies without either using larger regions of the visual field (reducing resolution) or having the stimulus spatial frequency be less well-defined (thus effectively re-introducing higher frequencies).

Mantises are visually guided predators with unusually good acuity among insects. Specifically, they have a central foveal region with greater acuity (Rossel 1979). We might, therefore, expect to find different results in other insects that lack foveae. We are not aware of similar behavioural experiments in other species. However, previous investigations into the neural basis of motion detection in houseflies have found that the motion-sensitive neurons saturated for small angular extents (~12°) of patterns but increased in response to increase in the oscillation amplitude of the pattern (Borst et al. 1995).

Our results have implications for the presentation of moving stimuli to insects on cylindrical or planar screens. Both of these have been previously used in experiments (Reichardt and Wenking 1969; Pick and Buchner 1979; Dvorak et al. 1980; Srinivasan and Dvorak 1980; Reichardt and Guo 1986; Nityananda et al. 2015), and simulate, respectively, rotation and sideways translation (perpendicular to the line of sight). In principle, the results obtained using both approaches could be different, since the speeds and spatial frequencies in the periphery would be different for these two presentations, as described above. Our results, however, show that in mantises, the periphery does not drive the optomotor response as strongly as the central visual field. Therefore, since the optic flow for both rotational and sideways translational movements (Fig. 1) is similar in the central region, we should not expect the major differences in response to stimuli presented on planar versus cylindrical screens. The spatial and temporal frequency tuning of the optomotor response in these two cases should also be comparable. This might not, however, be true for other animals where similar paradigms have been used. If peripheral stimuli do have a strong effect on the optomotor response in other animals, it would be important to choose the type of presentation used in vision studies carefully so that comparison across studies would be possible.

These combined results can be modelled with the elementary motion detectors (EMDs) known to underlie motion detection in insects (Hassenstein and Reichardt 1956; Reichardt and Egelhaaf 1988). In such a model, the EMDs would have a saturating response to contrast. This is a modification of the classical motion detection models used so far in the insect literature. The EMDs feed into lobula plate tangential neurons (Haag et al. 1999). The relevant neurons for our experiment would be the HS neurons that integrate motion horizontally rather than vertically (Hausen 1982a, b). There is also a saturating response to the total EMD input to the HS output. Our results further suggest that the full saturation response is reached when the EMDs detect a full-contrast central stimulus subtending an angle of around 85°. Further experiments would be necessary to confirm important aspects of this model in other insects including the saturation to contrast of the EMDs and the differential weighting of the input from peripheral and central ommatidia. These would shed further light on the mechanisms underlying spatial integration in the insect motion detection system.
